# Prioritization of patient safety health policies: Delphi survey using patient safety experts in Japan

**DOI:** 10.1371/journal.pone.0239179

**Published:** 2020-09-17

**Authors:** Yosuke Hatakeyama, Shigeru Fujita, Shuhei Iida, Yoji Nagai, Yoshiko Shimamori, Junko Ayuzawa, Tomohiro Hirao, Ryo Onishi, Kanako Seto, Kunichika Matsumoto, Tomonori Hasegawa

**Affiliations:** 1 Toho University School of Medicine, Tokyo, Japan; 2 All Japan Hospital Association, Tokyo, Japan; 3 Nerima General Hospital, Tokyo, Japan; 4 Institute for Healthcare Quality Improvement, Tokyo Healthcare Foundation, Tokyo, Japan; 5 Hitachinaka General Hospital, Hitachinaka, Ibaraki, Japan; 6 Iwate Medical University School of Nursing, Iwate, Japan; 7 Graduate School of Medical Sciences, Kyushu University, Fukuoka, Japan; 8 Faculty of Medicine, Kagawa University, Takamatsu, Kagawa, Japan; South China University of Technology, CHINA

## Abstract

Various patient safety interventions have been implemented since the late 1990s, but their evaluation has been lacking. To obtain basic information for prioritizing patient safety interventions, this study aimed to extract high-priority interventions in Japan and to identify the factors that influence the setting of priority. Six perspectives (contribution, dissemination, impact, cost, urgency, and priority) on 42 patient safety interventions classified into 3 levels (system, organizational, and clinical) were evaluated by Japanese experts using the Delphi technique. We examined the relationships of the levels and the perspectives on interventions with the transition of the consensus state in rounds 1 and 3. After extracting the high-priority interventions, a chi-squared test was used to examine the relationship of the levels and the impact/cost ratio with high priority. Regression models were used to examine the influence of each perspective on priority. There was a significant relationship between the level of interventions and the transition of the consensus state (p = 0.033). System-level interventions had a low probability of achieving consensus. “Human resources interventions,” “professional education and training,” “medication management/reconciliation protocols,” “pay-for performance (P4P) schemes and financing for safety,” “digital technology solutions to improve safety,” and “hand hygiene initiatives” were extracted as high-priority interventions. The level and the impact/cost ratio of interventions had no significant relationships with high priority. In the regression model, dissemination and impact had an influence on priority (β = -0.628 and 0.941, respectively; adjusted R-squared = 0.646). The influence of impact and dissemination on the priority of interventions suggests that it is important to examine the dissemination degree and impact of interventions in each country for prioritizing interventions.

## Introduction

Patient safety has been a global concern against the background of the occurrence of serious adverse events since the late 1990s [1]. The World Health Organization defined patient safety as “the reduction of risk of unnecessary harm associated with health care to an acceptable minimum” [2]. To improve patient safety, various activities have been introduced by governments, medical/specialty societies, accreditation bodies, and healthcare organizations in many countries, such as incident reporting, training and deployment of safety managers, standardization of care, and changes to payment schemes [3]. Recently, Hasegawa and Fujita reported on the current status of patient safety policies in the Organisation for Economic Co-operation and Development (OECD) member countries [4].

Japan experienced a series of serious patient safety accidents in the late 1990s. In 2002, the General Policy for Medical Safety was published by the Ministry of Health, Labour and Welfare. It stressed the importance of a systematic approach for securing safe workplace environments, and cultivating a patient safety culture. Hospitals were required to have safety standards, an in-hospital incident reporting system, a patient safety committee, and education for hospital staff about patient safety. Hospitals were encouraged to assign patient safety managers, and hospitals with patient safety managers could receive more money as of 2006. A nationwide adverse event reporting system was introduced (2004 for accidents, 2005 for close calls). It is one of the biggest databases of adverse events in the world. In 2009, a no-fault compensation system for cerebral palsy was introduced. Babies with medium to severe cerebral palsy can receive money from this insurance scheme. In 2015, a lethal adverse event investigation system was introduced. In this unique system, the hospital chief executive officer has to report any lethal case possibly due to an adverse event to a third-party organization (the Adverse Event Investigation and Support Center), conduct an in-hospital investigation, and submit the report along with preventive methods to the organization.

With increasing healthcare expenditure, prioritizing patient safety interventions based on evidence has become an important issue. However, previous studies have not found a consensus on whether patient safety interventions improve patient safety [5, 6]. A few individual patient safety projects have been largely successful, but it remains unclear if other efforts are effective in improving patient safety [7]. Given the cost of patient safety interventions, it is important to prioritize them in a limited resource environment; however, the published evidence on their cost and cost-effectiveness is insufficient [8].

The OECD published a report titled “The Economics of Patient Safety: Strengthening a Value-Based Approach to Reducing Patient Harm at National Level” in 2017 [9]. In this report, the OECD showed the results of a panel survey of expert academics and policy makers about prioritizing patient safety interventions in a resource-constrained environment. Based on experts’ ratings on the impact and implementation cost of patient safety interventions, they extracted valued interventions for improving patient safety from 42 interventions at the system, organizational, and clinical levels. Subsequently, they suggested that using a survey and nominal group technique or other approach (e.g., Delphi) for promoting value-based investment in patient safety could identify specific priority areas based on local contexts and expertise, and thus identify the optimal mix of components for a national patient safety strategy.

The OECD used the impact and cost of patient safety as factors to identify the priority of patient safety interventions. However, patient safety interventions to be promoted could be defined by not only the impact and cost but also other factors. It is important to identify factors that should be counted when deciding the priority of patient safety interventions in each country or region. It has not been made clear which factors should be considered in prioritizing patient safety interventions.

Based on patient safety experts’ consensus, this study aimed to extract high-priority patient safety interventions in Japan and to identify the factors influencing the priority of these interventions.

## Materials and methods

### Delphi process

The Delphi technique was used for obtaining a consensus on patient safety interventions from patient safety experts. This technique is a forecasting method that involves repeatedly asking experts to summarize their opinions [10, 11]. The Delphi technique has been used to solve an array of healthcare problems ranging from those of an individual hospital or department to those of a statewide agency or state [12].

We chose 24 experts including two representatives of nationwide organizations related to patient safety, five hospital administrators, seven patient safety managers at each hospital, eight researchers of patient safety and two other famous in this field. A Delphi survey was conducted over three rounds by mail (round 1) and e-mail (rounds 2 and 3) from July to October 2017. During the rounds, the results of the previous round were presented to the participants.

According to the OECD report [9], the questionnaire consisted of 10 patient safety interventions at the system level, 14 at the organizational level, and 18 at the clinical level (totaling 42 interventions) on a 5-point Likert scale. Table 1 shows the interventions included. We set 6 perspectives for assessing the importance of interventions in the past (contribution), present (dissemination), and future (impact, cost, urgency, and priority). In each round, participants were asked to rate all 42 interventions on a 5-point scale from 6 perspectives: past contribution to patient safety (contribution; 1: Small to 5: Large), present dissemination (dissemination; 1: Low to 5: High), expected effects in reducing harm if implemented (impact; 1: Low to 5: High), cost to implement (cost; 1: Low to 5: High), urgency (urgency; 1: Low to 5: High), and priority for future implementation (priority; 1: Low to 5: High). Ratings on past contributions were asked in round 1 only; the others were asked in all three rounds.

**Table 1:** 42 patient safety interventions.

Level	Intervention
System	
	Safety Standards
	Public reporting of patient safety indicators
	Mandatory reporting of specified adverse events
	Pay-for performance (P4P) schemes and financing for safety
	Professional education and training
	Electronic Health Record (EHR) systems
	No-fault medical harm compensation scheme
	System-level public engagement and health literacy initiatives
	Theme-based national safety initiatives
	A national agency responsible for patient safety
Organization	
	Clinical governance frameworks and systems for patient safety
	Clinical incident reporting and management system
	Integrated patient complaint- and incident-reporting
	Monitoring and feedback of patient safety indicators
	Patient-engagement initiatives
	Clinical communication protocols and training
	Digital technology solutions to improve safety
	Human resources interventions
	Building a positive safety culture
	Infection detection, reporting and surveillance systems
	Hand hygiene initiatives
	Antimicrobial stewardship
	Blood and blood product management protocols
	Medical equipment sterilisation protocols
Clinical	
	Medication management / reconciliation protocols
	Transcribing error systems and protocols
	Smart infusion pumps and drug administration systems
	Aseptic technique protocols and barrier precautions
	Urinary catheter use and insertion protocols
	Central venous catheter insertion protocols
	Ventilator-associated pneumonia minimisation protocols
	Procedural / surgical checklists
	Operating room integration and display technology
	Peri-operative medication protocols
	Venous thromboembolism (VTE) prevention protocols
	Clinical care standards
	Pressure injury (ulcer) prevention protocols
	Falls prevention initiatives
	Acute delirium & cognitive impairment management initiatives
	Response to clinical deterioration
	Patient hydration and nutrition standards
	Patient identification and procedure matching protocols

Ethical Approval was obtained from the Ethics Committee of Toho University School of Medicine (No. A17025).

### Data analysis

For assessing the variance of the scores rated by experts, we calculated the means (with standard deviations [SD]) and medians (with interquartile range [IQR]) of the rating scores for each intervention. To assess the convergence through the rounds, we examined the relationship between the level (system, organizational, and clinical) of interventions and the transition of the consensus state in rounds 1 and 3. We defined the consensus as being reached if IQR = < 1 following the previous studies using 5-point Likert scale [13, 14]. The transitions of the consensus state were classified into four categories: 1) consistent consensus (consensus was achieved in both round 1 and round 3), 2) change to consensus (consensus was not achieved in round 1, but achieved in round 3), 3) change to dissensus (consensus was achieved in round 1, but not achieved in round 3), and 4) consistent dissensus (consensus was not achieved in round 1 or round 3). We used a chi-squared test for assessing the independence of the relationship between the levels of interventions and the consensus state from round 1 to 3. If relevance was found, we calculated the adjusted residuals of each cell in a cross table as a post hoc analysis. Consequently, the relationships between the five perspectives and the transitions of the consensus state were examined using the same methods.

A high-priority intervention was defined as an intervention with a median above the median of all interventions’ priority. To examine the relationships of the levels and the impact/cost ratio of interventions with the high-priority interventions, we calculated the mean and median scores of 42 interventions in each perspective using the results of the contributions in round 1 and the others in round 3. A chi-squared test for independence between the priority of interventions (high priority or not high priority) and the level of interventions (system, organizational, or clinical) was conducted. Subsequently, after defining high-impact/cost ratio interventions as interventions with a median above the median of all interventions in impact/cost ratio, we performed a chi-squared test for independence between priority (high priority or not high priority) and the impact/cost ratio (high impact/cost ratio or low impact/cost ratio).

Spearman’s correlation coefficients (ρ) were calculated from the mean values of each item in each perspective to assess the relationships within the six perspectives. These correlation coefficients represent the relationship of perspectives with the priority including the influence of the other perspectives. To identify the influences of each perspective on priority, we examined the impact on priority from the other perspectives with regression models adjusted for the influence of each perspective. We set four models. Model 1 was a model for examining the impact and cost of interventions on their priority based on the OECD report. Model 2 included contribution and dissemination as explanatory variables and was performed to assess the influence of past and present patient safety efforts on the priority of interventions. Including contribution, dissemination, and impact, we assessed the influence of previous efforts and the impact of interventions in Model 3. Model 4 was conducted to examine the influence of all perspectives on the priority of interventions with simultaneous forced entry.

All data were analyzed using IBM SPSS Statistics version 25, and a p value of <0.05 was considered statistically significant.

## Results

### Delphi process

All 24 experts (Table 2) responded in all 3 rounds. The results of rounds 1 and 3 are shown in S1 Table.

**Table 2:** Baseline characteristics of experts.

		n	%
Domain			
	Representative of Nationwide Organization related to Patient Safety	2	8.3
	Hospital Administrator	5	20.8
	Patient Safety Manager	7	29.2
	Researcher of Patient Safety	8	33.3
	Other	2	8.3
Gender			
	Male	18	75.0
	Female	6	25.0
Profession			
	Doctor	15	62.5
	Nurse	4	16.7
	Pharmacist	2	8.3
	Other	3	12.5

Table 3 shows the relationship between the levels of interventions and the transition of the consensus state from round 1 to 3 in the Delphi survey. Each cell shows the numbers and percentages of items (n, %), adjusted residuals (Adj. Res), and p values of adjusted residuals (p). There was a significant relationship between the levels of interventions and the transition of the consensus state (chi-squared = 13.723, degree of freedom [df] = 6, p = 0.033). In the residual analysis, we found a tendency for dissensus in priority of interventions at the system level (adjusted residual = 2.62, p = 0.009) and a tendency for consensus in priority of them at the clinical level (adjusted residual = 2.43, p = 0.015). There were no significant relationships between the six perspectives and the transition of the consensus state (chi-squared = 1.625, df = 4, p = 0.804).

**Table 3:** Relationship between the level of interventions and the transition of the consensus in rounds 1 and 3.

Level		Consistent consensus	Change to consensus	Change to dissensus	Consistent dissensus	Total
System	n	27	18	0	5	50
	%	54.0%	36.0%	0.0%	10.0%	100.0%
	Adj. Res	-2.36	1.42	-0.56	2.62	
	p	0.018	0.154	0.575	0.009	
Organizational	n	46	21	1	2	70
	%	65.7%	30.0%	1.4%	2.9%	100.0%
	Adj. Res	-0.42	0.43	1.42	-0.51	
	p	0.677	0.664	0.156	0.610	
Clinical	n	69	20	0	1	90
	%	76.7%	22.2%	0.0%	1.1%	100.0%
	Adj. Res	2.43	-1.64	-0.87	-1.77	
	p	0.015	0.101	0.385	0.077	
Total	n	142	59	1	8	210
	%	67.6%	28.1%	0.5%	3.8%	100.0%

Chi-squared was 13.723 (p = 0.033). Abbreviations: Adj. Res = Adjusted Residuals.

### Relationship of the level and the impact/cost ratio of interventions with high-priority interventions

The median score of the 42 interventions on priority was 3.61. Of the 42 interventions, 21 were extracted as high-priority interventions. Table 4 shows the high-priority interventions with their level and the ratio of impact/cost. There were 2 interventions from the system level, 8 from the organizational level, and 11 from the clinical level. No significant relationship between the priority of interventions (high priority or not) and the level of interventions was observed (chi-squared = 4.755, df = 2, p = 0.092).

**Table 4:** Priority, impact, and cost of interventions (ordered by priority).

High-Priority Interventions	Level	Priority	Impact	Cost	Impact/Cost Ratio
Human resources interventions	Organizational	4.25	3.83	4.33	0.88
Professional education and training	System	4.25	3.92	3.38	1.16
Medication management/ reconciliation protocols	Clinical	4.22	4.13	3.57	1.16
Pay-for performance (P4P) schemes and financing for safety	System	4.08	3.83	3.75	1.02
Digital technology solutions to improve safety	Organizational	4.04	4.04	4.71	0.86
Hand hygiene initiatives	Organizational	4.00	4.08	3.13	1.30
Transcribing error systems and protocols	Clinical	3.96	4.00	3.75	1.07
Response to clinical deterioration	Clinical	3.91	3.83	3.48	1.10
Acute delirium & cognitive impairment management initiatives	Clinical	3.91	3.65	3.00	1.22
Clinical communication protocols and training	Organizational	3.91	3.87	3.04	1.27
Patient identification and procedure matching protocols	Clinical	3.79	3.92	2.54	1.54
Procedural/surgical checklists	Clinical	3.79	3.96	2.79	1.42
Antimicrobial stewardship	Organizational	3.79	3.88	3.08	1.26
Venous thromboembolism (VTE) prevention protocols	Clinical	3.78	3.96	3.35	1.18
Peri-operative medication protocols	Clinical	3.74	3.87	2.91	1.33
Central venous catheter insertion protocols	Clinical	3.71	4.00	3.63	1.10
Infection detection, reporting, and surveillance systems	Organizational	3.71	3.92	3.58	1.09
Clinical governance frameworks and systems for patient safety	Organizational	3.67	3.75	3.13	1.20
Clinical care standards	Clinical	3.65	3.74	3.04	1.23
Patient-engagement initiatives	Organizational	3.65	3.61	3.00	1.20
Aseptic technique protocols and barrier precautions	Clinical	3.63	4.00	3.96	1.01

Of the 21 high-priority interventions, 2 interventions—“human resources interventions” (ratio of impact/cost = 0.88) and “digital technology solutions to improve safety” (ratio of impact/cost = 0.86)—were low cost-effective. The other 19 interventions with high priority were evaluated as high cost-effective: “patient identification and procedure matching protocols” (ratio of impact/cost = 1.54), “procedural/surgical checklists” (ratio of impact/cost = 1.42), “peri-operative medication protocols” (ratio of impact/cost = 1.33), and “hand hygiene initiatives” (ratio of impact/cost = 1.30) were among them. There was no significant relationship between the impact/cost ratio and high priority (chi-squared = 2.381, df = 1, p = 0.123).

### Influence of perspectives on priority

Table 5 shows the item scores by the level of intervention based on the results of the contribution in round 1 and the others in round 3. The mean scores of contribution, dissemination, impact, urgency, and priority were higher in the order of clinical, organizational, and system levels. As for cost, the mean scores were in the reverse order of the other perspectives. The standard deviations of each perspective were in the order of system, organizational, and clinical levels in all perspectives.

**Table 5:** Scores by the level of interventions in each perspective.

Perspective	Level	Mean	SD	Median	IQR
Contribution	All	3.10	0.39	3.14	0.65
	System	2.96	0.52	3.09	0.84
	Organizational	3.10	0.40	2.94	0.81
	Clinical	3.18	0.30	3.17	0.51
Dissemination	All	3.17	0.54	3.15	0.85
	System	2.84	0.61	2.96	1.11
	Organizational	3.19	0.59	3.06	1.12
	Clinical	3.33	0.38	3.28	0.75
Impact	All	3.72	0.27	3.79	0.32
	System	3.56	0.28	3.61	0.49
	Organizational	3.75	0.27	3.81	0.29
	Clinical	3.80	0.23	3.83	0.31
Cost	All	3.43	0.53	3.31	0.65
	System	3.74	0.57	3.61	0.89
	Organizational	3.33	0.55	3.13	0.45
	Clinical	3.33	0.46	3.31	0.66
Urgency	All	3.49	0.32	3.56	0.54
	System	3.32	0.37	3.19	0.60
	Organizational	3.50	0.33	3.51	0.47
	Clinical	3.56	0.27	3.62	0.44
Priority	All	3.59	0.36	3.61	0.50
	System	3.45	0.41	3.38	0.52
	Organizational	3.63	0.38	3.66	0.50
	Clinical	3.63	0.32	3.68	0.37

Scores for contribution were calculated from the results of round 1, and the others from those of round 3.

Abbreviations: SD = Standard Deviation, IQR = Interquartile Range.

Table 6 shows the correlations between the six perspectives. There was a strong relationship between contribution and dissemination (ρ = 0.904), but neither of the two perspectives did not have significant correlations with priority. Impact had moderate correlations with priority (ρ = 0.719). There was a considerably strong correlation between urgency and priority (ρ = 0.960). These results demonstrated that experts considered the future importance (impact, urgency, and priority) of interventions to be different from the past and present importance (contribution and dissemination) of them.

**Table 6:** Correlation between the six perspectives (Spearman’s correlation coefficients).

	Contribution		Dissemination		Impact		Cost	Urgency		Priority
Contribution	1.000									
Dissemination	0.904	***	1.000							
Impact	0.502	***	0.476	**	1.000					
Cost	0.036		-0.089		0.189	**	1.000			
Urgency	0.072		-0.017		0.727	***	0.205	1.000		
Priority	0.074		-0.008		0.719	***	0.153	0.960	***	1.000

***p<0.001

**p<0.01

*p<0.05.

Based on the results of the correlation analysis, we excluded urgency from the regression analysis because urgency and priority might be considered the same perspectives in the panels. The results of the regression model with priority as the objective variable and the other four perspectives as explanatory variables are shown in Table 7. In Model 1, which assessed the influence of impact and cost as used in the OECD report,^9^ impact had an influence on priority (standardized coefficient (β) = 0.710). In Model 2, with contribution and dissemination of previous interventions, there was no significant influence on priority. In Model 3, adding impact to the perspectives used in Model 2, dissemination had a negative correlation (β = -0.504) and impact a positive one (β = -0.893) with priority. In Model 4, using four perspectives, dissemination had a negative correlation (β = -0.628) and impact a positive one (β = 0.941) with priority. Cost had no significant influence on priority in any of these models. The highest adjusted R-squared was observed in Model 4 (0.646), followed by Model 3 (0.639) and Model 1 (0.509). The regression analysis indicated that dissemination as well as impact was a factor should be considered in prioritizing interventions.

**Table 7:** The influence of contribution, dissemination, impact, and cost on priority (standardized coefficients).

Perspectives	Model 1		Model 2	Model 3		Model 4	
Contribution			0.600	0.103		0.177	
Dissemination			-0.548	-0.504	*	-0.628	**
Impact	0.710	***		0.893	***	0.941	***
Cost	0.060					-0.141	
Adj. R-Squared	0.509		0.030	0.639		0.646	

Objective variables: Priority.

***p<0.001

**p<0.01

*p<0.05.

## Discussion

By using the Delphi technique to summarize the opinions of patient safety experts, this study extracted high-priority patient safety interventions and examined the influence of perspectives on the priority of patient safety interventions.

In the Delphi process, the probability of a transition state of consistent consensus was lower and the probability of a transition of consistent dissensus was higher than expected in system-level interventions. Similarly, there were large SDs and IQRs for scores at the system level in each perspective. These results might mean that the opinions of the experts on the system-level interventions showed diversity and difficulty in achieving a consensus compared to the organizational and clinical levels. In the OECD report, the largest SD of cost and impact was found at the system level: “pay-for performance (P4P) schemes for patient safety” for cost and “system-level public engagement and health literacy initiatives” for impact [9]. It might have been difficult for the experts to have the same images on system-level interventions since some experts were working on the clinical and organizational levels.

The OECD report extracted valued interventions based on the ratio of impact/cost [9]. However, the priority of interventions was not related to the impact/cost ratio in the present study. The ratio of impact/cost could be reworded as cost-effectiveness. Cost-effectiveness analysis, such as quality adjusted life year and the incremental cost-effectiveness ratio, has been introduced for approving new drugs in several countries, such as the United Kingdom, Canada, and Australia [15]. In Japan, cost-effectiveness analysis for the pricing of drugs and medical devices started in the 2017 fiscal year [16]. Japanese experts might not have been accustomed to associating the impact/cost ratio with the priority of intervention, as in this study, since cost-effectiveness analysis in Japan has only just begun and has not been widely disseminated.

In the regression models, it was revealed that impact had a significant positive influence and dissemination a significant negative influence on priority. While the OECD report identified the valued interventions from the impact/cost ratio [9], this study suggested that, adding to the impact of interventions, the degree of dissemination of the interventions was also important in deciding the priority of patient safety interventions. The dissemination of patient safety interventions could vary among countries or regions. Hasegawa and Fujita reported the variety in the adoption of patient safety policies in OECD member counties [4]. The difference in the degree of dissemination of patient safety interventions would be derived from the history and system of the healthcare in each country or region. For example, “clinical incident reporting and management system” had the highest score for contribution (4.08) and the lowest score for priority (3.29, lower than the first quartile of 42 interventions) in this study. As mentioned, since 2002, all hospitals have been required by law to have an in-hospital incident reporting system, and the nationwide incident reporting system began in 2004 in Japan. These already introduced health policies might lessen the priority of a “clinical incident reporting and management system.” The results of this study do not necessarily deny the recommendations of previous studies; rather, it is necessary to examine the dissemination degree and impact of the recommended interventions before applying them in a specified country or region.

When using the Delphi technique, we asked 24 experts, and used IQR as the indicator of consensus. There is no consensus on “an optimal number of subjects in a Delphi study” [17]. In a systematic review on Delphi studies showing the widely varied numbers of participants, 40.0% studies used 11–25 participants in final round [18]. In addition, “Delphi subjects should be highly trained and competent within the specialized area of knowledge related to the target issue” [17]. We selected 24 experts who were representatives of nationwide organizations related to patient safety, hospital administrators, patient safety managers at each hospital, researchers of patient safety and other famous in this field. Diamond et al. conducted a systematic review of 72 Delphi studies, and reported that the definition of consensus showed a wide range of definitions among studies. The most common definition for consensus was percent agreement (percentage of same rating, used in 25 studies), followed by the proportion of ratings within a range (percentage of rating within a certain range, used in 16 studies), the measure of central tendency (median ranking, in 8 studies), and decrease in variance (interquartile range, 6 studies) [18]. Percent agreement and proportion of agreement were likely to be used in dichotomous questions. IQR was used in this study since 5-point Likert rating scale was used for assessing importance of the patient safety interventions.

This study has certain limitations. The results might be influenced from other factors that we did not examined, such as the order of Delphi questionnaire and the characteristics of experts [19]. The priority of the interventions presented in this study was based on the results of a Delphi survey with Japanese experts in patient safety. Therefore, the results should be applied to other countries and regions with caution.

## Conclusion

Using the Delphi technique, “human resources interventions,” “professional education and training,” “medication management/reconciliation protocols,” “pay-for performance (P4P) schemes and financing for safety,” “digital technology solutions to improve safety,” and “hand hygiene initiatives” were extracted as high-priority interventions. It was suggested that the experts in patient safety had difficulty assessing interventions at the system level and the cost-effectiveness of the interventions. The ratio of impact/cost, which was used in the OECD report, and level of interventions did not have a significant relationship with priority. Instead, a positive influence of impact and a negative influence of dissemination on priority were found. The influence of impact and dissemination on the priority of patient safety interventions might suggest that it is important to examine the dissemination degree and impact of interventions in each country when prioritizing interventions.

## Supporting information

S1 Table Item scores of each intervention in round 1 and 3. (DOCX)
