# Supplementary material for: Prioritization of patient safety health policies: Delphi survey using patient safety experts in Japan
Source: PLoS One. 2020 Sep 17;15(9):e0239179. doi: 10.1371/journal.pone.0239179 (PMC7497979; doi:10.1371/journal.pone.0239179)
Supplement: S1 Table — (DOCX) [file pone.0239179.s001.docx]

**S1 Table.** **Item scores of each intervention in round 1 and 3.**

| Level | Intervention/  Perspective | | Round 1 | | | |  | Round 3 | | | |
| --- | --- | --- | --- | --- | --- | --- | --- | --- | --- | --- | --- |
|  |  |  | Mean | SD | Median | IQR |  | Mean | SD | Median | IQR |
| System | Safety Standards | |  |  |  |  |  |  |  |  |  |
|  |  | Contribution | 3.46 | 1.06 | 4.00 | 1.00 |  |  |  |  |  |
|  |  | Dissemination | 3.63 | 1.01 | 4.00 | 1.00 |  | 3.75 | 0.68 | 4.00 | 1.00 |
|  |  | Impact | 3.63 | 0.82 | 4.00 | 1.00 |  | 3.79 | 0.72 | 4.00 | 1.00 |
|  |  | Cost | 3.79 | 0.98 | 4.00 | 2.00 |  | 4.13 | 0.80 | 4.00 | 1.00 |
|  |  | Urgency | 2.79 | 0.98 | 3.00 | 1.75 |  | 2.96 | 0.75 | 3.00 | 0.00 |
|  |  | Priority | 3.29 | 0.86 | 3.00 | 1.00 |  | 3.17 | 0.87 | 3.00 | 1.00 |
| System | Public reporting of patient safety indicators | | | | |  |  |  |  |  |  |
|  |  | Contribution | 2.74 | 0.86 | 3.00 | 1.00 |  |  |  |  |  |
|  |  | Dissemination | 2.61 | 0.84 | 3.00 | 1.00 |  | 2.70 | 0.70 | 3.00 | 1.00 |
|  |  | Impact | 3.22 | 0.80 | 3.00 | 1.00 |  | 3.26 | 0.54 | 3.00 | 1.00 |
|  |  | Cost | 3.30 | 0.63 | 3.00 | 1.00 |  | 3.43 | 0.59 | 3.00 | 1.00 |
|  |  | Urgency | 2.91 | 1.00 | 3.00 | 2.00 |  | 3.00 | 0.52 | 3.00 | 0.00 |
|  |  | Priority | 3.17 | 0.78 | 3.00 | 1.00 |  | 3.17 | 0.72 | 3.00 | 0.00 |
| System | Mandatory reporting of specified adverse events | | | | | |  |  |  |  |  |
|  |  | Contribution | 3.17 | 1.09 | 3.00 | 1.75 |  |  |  |  |  |
|  |  | Dissemination | 3.21 | 1.06 | 3.00 | 1.75 |  | 3.21 | 0.51 | 3.00 | 0.75 |
|  |  | Impact | 3.42 | 0.83 | 3.00 | 1.00 |  | 3.33 | 0.82 | 3.00 | 1.00 |
|  |  | Cost | 3.13 | 0.85 | 3.00 | 1.00 |  | 3.46 | 0.51 | 3.00 | 1.00 |
|  |  | Urgency | 3.08 | 0.93 | 3.00 | 0.75 |  | 3.08 | 0.72 | 3.00 | 0.75 |
|  |  | Priority | 3.33 | 1.01 | 3.00 | 1.75 |  | 3.42 | 0.83 | 3.50 | 1.00 |
| System | Pay-for performance (P4P) schemes and financing for safety | | | | | | |  |  |  |  |
|  |  | Contribution | 3.42 | 1.25 | 3.00 | 1.75 |  |  |  |  |  |
|  |  | Dissemination | 3.38 | 1.31 | 3.00 | 1.75 |  | 3.13 | 0.95 | 3.00 | 2.00 |
|  |  | Impact | 4.21 | 0.66 | 4.00 | 1.00 |  | 3.83 | 0.64 | 4.00 | 0.00 |
|  |  | Cost | 3.54 | 1.10 | 3.00 | 1.75 |  | 3.75 | 0.74 | 4.00 | 1.00 |
|  |  | Urgency | 3.96 | 0.81 | 4.00 | 2.00 |  | 3.79 | 0.72 | 4.00 | 1.00 |
|  |  | Priority | 3.96 | 0.81 | 4.00 | 2.00 |  | 4.08 | 0.78 | 4.00 | 1.75 |
| System | Professional education and training | | | |  |  |  |  |  |  |  |
|  |  | Contribution | 3.50 | 1.06 | 4.00 | 1.00 |  |  |  |  |  |
|  |  | Dissemination | 3.25 | 0.90 | 3.00 | 1.75 |  | 3.46 | 0.72 | 3.50 | 1.00 |
|  |  | Impact | 4.00 | 0.78 | 4.00 | 2.00 |  | 3.92 | 0.65 | 4.00 | 0.75 |
|  |  | Cost | 3.04 | 0.86 | 3.00 | 1.00 |  | 3.38 | 0.65 | 3.00 | 1.00 |
|  |  | Urgency | 3.71 | 1.00 | 4.00 | 1.75 |  | 4.00 | 0.66 | 4.00 | 0.00 |
|  |  | Priority | 4.04 | 0.95 | 4.00 | 2.00 |  | 4.25 | 0.61 | 4.00 | 1.00 |
| System | Electronic Health Record (EHR) systems | | | | |  |  |  |  |  |  |
|  |  | Contribution | 2.13 | 0.95 | 2.00 | 0.00 |  |  |  |  |  |
|  |  | Dissemination | 1.96 | 0.69 | 2.00 | 0.75 |  | 2.13 | 0.54 | 2.00 | 0.00 |
|  |  | Impact | 3.67 | 1.13 | 4.00 | 2.00 |  | 3.71 | 1.08 | 4.00 | 1.75 |
|  |  | Cost | 4.54 | 0.66 | 5.00 | 1.00 |  | 4.58 | 0.50 | 5.00 | 1.00 |
|  |  | Urgency | 3.38 | 1.06 | 3.00 | 1.00 |  | 3.50 | 0.78 | 3.50 | 1.00 |
|  |  | Priority | 3.71 | 1.12 | 4.00 | 2.00 |  | 3.54 | 0.88 | 4.00 | 1.00 |
| System | No-fault medical harm compensation scheme | | | | |  |  |  |  |  |  |
|  |  | Contribution | 2.75 | 1.15 | 2.50 | 2.00 |  |  |  |  |  |
|  |  | Dissemination | 2.46 | 1.02 | 2.00 | 1.00 |  | 1.88 | 0.54 | 2.00 | 0.00 |
|  |  | Impact | 3.46 | 1.06 | 3.50 | 1.00 |  | 3.42 | 0.88 | 3.50 | 1.00 |
|  |  | Cost | 4.13 | 0.85 | 4.00 | 2.00 |  | 4.46 | 0.59 | 4.50 | 1.00 |
|  |  | Urgency | 3.29 | 1.08 | 3.50 | 1.00 |  | 3.54 | 0.78 | 4.00 | 1.00 |
|  |  | Priority | 3.33 | 1.09 | 3.00 | 1.00 |  | 3.42 | 0.78 | 3.00 | 1.00 |
| System | System-level public engagement and health literacy initiatives | | | | | | | |  |  |  |
|  |  | Contribution | 2.13 | 0.95 | 2.00 | 1.75 |  |  |  |  |  |
|  |  | Dissemination | 2.00 | 0.72 | 2.00 | 1.50 |  | 2.17 | 0.48 | 2.00 | 0.00 |
|  |  | Impact | 3.04 | 1.16 | 3.00 | 2.00 |  | 3.08 | 0.88 | 3.00 | 1.50 |
|  |  | Cost | 2.96 | 0.95 | 3.00 | 1.00 |  | 2.88 | 0.68 | 3.00 | 0.75 |
|  |  | Urgency | 3.04 | 1.00 | 3.00 | 2.00 |  | 3.00 | 0.83 | 3.00 | 1.75 |
|  |  | Priority | 3.04 | 1.12 | 3.00 | 2.00 |  | 3.00 | 0.72 | 3.00 | 0.00 |
| System | Theme-based national safety initiatives | | | | |  |  |  |  |  |  |
|  |  | Contribution | 3.00 | 1.10 | 3.00 | 2.00 |  |  |  |  |  |
|  |  | Dissemination | 3.33 | 0.82 | 3.00 | 1.00 |  | 3.04 | 0.62 | 3.00 | 0.00 |
|  |  | Impact | 3.54 | 0.78 | 3.00 | 1.00 |  | 3.50 | 0.78 | 4.00 | 1.00 |
|  |  | Cost | 3.08 | 0.65 | 3.00 | 0.00 |  | 3.17 | 0.56 | 3.00 | 0.75 |
|  |  | Urgency | 3.08 | 0.83 | 3.00 | 0.00 |  | 3.08 | 0.50 | 3.00 | 0.00 |
|  |  | Priority | 3.08 | 0.97 | 3.00 | 0.00 |  | 3.13 | 0.54 | 3.00 | 0.00 |
| System | A national agency responsible for patient safety | | | | | |  |  |  |  |  |
|  |  | Contribution | 3.33 | 1.17 | 3.50 | 2.00 |  |  |  |  |  |
|  |  | Dissemination | 3.17 | 0.87 | 3.00 | 1.75 |  | 2.88 | 0.61 | 3.00 | 0.75 |
|  |  | Impact | 3.71 | 1.00 | 4.00 | 1.00 |  | 3.75 | 0.61 | 4.00 | 0.75 |
|  |  | Cost | 3.88 | 1.08 | 4.00 | 2.00 |  | 4.13 | 0.80 | 4.00 | 1.00 |
|  |  | Urgency | 3.13 | 0.99 | 3.00 | 1.75 |  | 3.29 | 0.62 | 3.00 | 1.00 |
|  |  | Priority | 3.33 | 1.13 | 3.50 | 1.00 |  | 3.33 | 0.64 | 3.00 | 1.00 |
| Organizational | Clinical governance frameworks and systems for patient safety | | | | | | | |  |  |  |
|  |  | Contribution | 2.88 | 1.03 | 3.00 | 1.75 |  |  |  |  |  |
|  |  | Dissemination | 3.21 | 0.98 | 3.00 | 2.00 |  | 2.92 | 0.72 | 3.00 | 1.00 |
|  |  | Impact | 3.96 | 0.86 | 4.00 | 0.75 |  | 3.75 | 0.61 | 4.00 | 0.00 |
|  |  | Cost | 2.92 | 0.72 | 3.00 | 1.00 |  | 3.13 | 0.61 | 3.00 | 0.75 |
|  |  | Urgency | 3.71 | 1.00 | 4.00 | 1.75 |  | 3.58 | 0.78 | 4.00 | 1.00 |
|  |  | Priority | 3.83 | 0.96 | 4.00 | 2.00 |  | 3.67 | 0.92 | 4.00 | 0.75 |
| Organizational | Clinical incident reporting and management system | | | | | |  |  |  |  |  |
|  |  | Contribution | 3.63 | 1.06 | 4.00 | 1.00 |  |  |  |  |  |
|  |  | Dissemination | 4.08 | 0.83 | 4.00 | 1.00 |  | 4.08 | 0.72 | 4.00 | 1.00 |
|  |  | Impact | 3.71 | 0.81 | 3.50 | 1.00 |  | 3.83 | 0.56 | 4.00 | 0.75 |
|  |  | Cost | 3.08 | 0.50 | 3.00 | 0.00 |  | 3.04 | 0.46 | 3.00 | 0.00 |
|  |  | Urgency | 3.04 | 1.00 | 3.00 | 2.00 |  | 3.21 | 0.59 | 3.00 | 1.00 |
|  |  | Priority | 3.33 | 1.01 | 3.00 | 1.00 |  | 3.29 | 0.55 | 3.00 | 1.00 |
| Organizational | Integrated patient complaint- and incident-reporting | | | | | |  |  |  |  |  |
|  |  | Contribution | 2.79 | 0.93 | 3.00 | 1.00 |  |  |  |  |  |
|  |  | Dissemination | 3.13 | 1.12 | 3.00 | 2.00 |  | 2.83 | 0.78 | 3.00 | 1.00 |
|  |  | Impact | 2.83 | 0.82 | 3.00 | 1.00 |  | 2.96 | 0.82 | 3.00 | 1.00 |
|  |  | Cost | 2.50 | 0.83 | 3.00 | 1.00 |  | 2.74 | 0.69 | 3.00 | 0.00 |
|  |  | Urgency | 2.71 | 0.86 | 3.00 | 1.00 |  | 2.91 | 0.60 | 3.00 | 0.00 |
|  |  | Priority | 2.75 | 0.79 | 3.00 | 1.00 |  | 2.78 | 0.52 | 3.00 | 1.00 |
| Organizational | Monitoring and feedback of patient safety indicators | | | | | |  |  |  |  |  |
|  |  | Contribution | 2.70 | 0.93 | 3.00 | 1.00 |  |  |  |  |  |
|  |  | Dissemination | 2.78 | 1.00 | 3.00 | 2.00 |  | 2.70 | 0.76 | 3.00 | 1.00 |
|  |  | Impact | 3.61 | 0.94 | 4.00 | 1.00 |  | 3.57 | 0.59 | 4.00 | 1.00 |
|  |  | Cost | 3.04 | 0.64 | 3.00 | 0.00 |  | 3.22 | 0.67 | 3.00 | 1.00 |
|  |  | Urgency | 3.43 | 0.95 | 3.00 | 1.00 |  | 3.30 | 0.47 | 3.00 | 1.00 |
|  |  | Priority | 3.48 | 0.90 | 4.00 | 1.00 |  | 3.48 | 0.59 | 4.00 | 1.00 |
| Organizational | Patient-engagement initiatives | | | |  |  |  |  |  |  |  |
|  |  | Contribution | 2.52 | 1.04 | 2.00 | 1.00 |  |  |  |  |  |
|  |  | Dissemination | 2.57 | 0.95 | 3.00 | 1.00 |  | 2.39 | 0.50 | 2.00 | 1.00 |
|  |  | Impact | 3.87 | 0.76 | 4.00 | 1.00 |  | 3.61 | 0.66 | 4.00 | 1.00 |
|  |  | Cost | 2.96 | 0.77 | 3.00 | 2.00 |  | 3.00 | 0.60 | 3.00 | 0.00 |
|  |  | Urgency | 3.48 | 0.95 | 4.00 | 1.00 |  | 3.35 | 0.65 | 3.00 | 1.00 |
|  |  | Priority | 3.61 | 0.94 | 4.00 | 1.00 |  | 3.65 | 0.78 | 4.00 | 1.00 |
| Organizational | Clinical communication protocols and training | | | | |  |  |  |  |  |  |
|  |  | Contribution | 2.88 | 1.12 | 3.00 | 1.75 |  |  |  |  |  |
|  |  | Dissemination | 2.92 | 0.88 | 3.00 | 1.75 |  | 2.70 | 0.63 | 3.00 | 1.00 |
|  |  | Impact | 4.00 | 0.74 | 4.00 | 0.00 |  | 3.87 | 0.46 | 4.00 | 0.00 |
|  |  | Cost | 3.04 | 0.93 | 3.00 | 2.00 |  | 3.04 | 0.47 | 3.00 | 0.00 |
|  |  | Urgency | 3.78 | 0.74 | 4.00 | 1.00 |  | 3.74 | 0.45 | 4.00 | 1.00 |
|  |  | Priority | 3.96 | 0.77 | 4.00 | 0.00 |  | 3.91 | 0.29 | 4.00 | 0.00 |
| Organizational | Digital technology solutions to improve safety | | | | |  |  |  |  |  |  |
|  |  | Contribution | 2.96 | 1.16 | 3.00 | 2.00 |  |  |  |  |  |
|  |  | Dissemination | 3.17 | 0.87 | 3.00 | 1.75 |  | 3.25 | 0.74 | 3.00 | 1.00 |
|  |  | Impact | 3.96 | 0.98 | 4.00 | 2.00 |  | 4.04 | 0.81 | 4.00 | 1.00 |
|  |  | Cost | 4.74 | 0.45 | 5.00 | 1.00 |  | 4.71 | 0.46 | 5.00 | 1.00 |
|  |  | Urgency | 3.96 | 1.11 | 4.00 | 2.00 |  | 4.08 | 0.88 | 4.00 | 1.00 |
|  |  | Priority | 4.13 | 1.01 | 4.00 | 2.00 |  | 4.04 | 0.86 | 4.00 | 1.00 |
| Organizational | Human resources interventions | | | |  |  |  |  |  |  |  |
|  |  | Contribution | 2.75 | 0.85 | 3.00 | 1.00 |  |  |  |  |  |
|  |  | Dissemination | 2.63 | 0.82 | 2.50 | 1.00 |  | 2.33 | 0.64 | 2.00 | 1.00 |
|  |  | Impact | 3.96 | 0.98 | 4.00 | 2.00 |  | 3.83 | 0.64 | 4.00 | 1.00 |
|  |  | Cost | 4.35 | 1.03 | 5.00 | 1.00 |  | 4.33 | 0.82 | 4.50 | 1.00 |
|  |  | Urgency | 4.04 | 1.07 | 4.00 | 2.00 |  | 3.96 | 0.69 | 4.00 | 0.75 |
|  |  | Priority | 4.04 | 1.02 | 4.00 | 2.00 |  | 4.25 | 0.74 | 4.00 | 1.00 |
| Organizational | Building a positive safety culture | | | |  |  |  |  |  |  |  |
|  |  | Contribution | 2.92 | 1.06 | 3.00 | 2.00 |  |  |  |  |  |
|  |  | Dissemination | 3.21 | 0.93 | 3.00 | 1.00 |  | 3.08 | 0.65 | 3.00 | 0.00 |
|  |  | Impact | 3.83 | 0.76 | 4.00 | 1.00 |  | 3.58 | 0.58 | 4.00 | 1.00 |
|  |  | Cost | 3.04 | 0.69 | 3.00 | 0.75 |  | 3.08 | 0.41 | 3.00 | 0.00 |
|  |  | Urgency | 3.46 | 0.88 | 3.00 | 1.00 |  | 3.29 | 0.55 | 3.00 | 1.00 |
|  |  | Priority | 3.71 | 0.81 | 4.00 | 1.00 |  | 3.58 | 0.50 | 4.00 | 1.00 |
| Organizational | Infection detection, reporting and surveillance systems | | | | | |  |  |  |  |  |
|  |  | Contribution | 3.67 | 1.13 | 4.00 | 1.75 |  |  |  |  |  |
|  |  | Dissemination | 3.79 | 1.10 | 4.00 | 2.00 |  | 3.79 | 0.51 | 4.00 | 0.75 |
|  |  | Impact | 3.83 | 0.82 | 4.00 | 1.00 |  | 3.92 | 0.50 | 4.00 | 0.00 |
|  |  | Cost | 3.63 | 0.71 | 4.00 | 1.00 |  | 3.58 | 0.50 | 4.00 | 1.00 |
|  |  | Urgency | 3.54 | 1.02 | 3.50 | 1.00 |  | 3.67 | 0.64 | 4.00 | 1.00 |
|  |  | Priority | 3.54 | 1.10 | 3.50 | 1.75 |  | 3.71 | 0.55 | 4.00 | 1.00 |
| Organizational | Hand hygiene initiatives | | |  |  |  |  |  |  |  |  |
|  |  | Contribution | 3.42 | 1.06 | 3.00 | 1.00 |  |  |  |  |  |
|  |  | Dissemination | 3.54 | 1.02 | 3.50 | 1.00 |  | 3.63 | 0.71 | 4.00 | 1.00 |
|  |  | Impact | 4.04 | 0.69 | 4.00 | 0.75 |  | 4.08 | 0.58 | 4.00 | 0.00 |
|  |  | Cost | 2.75 | 0.94 | 3.00 | 1.75 |  | 3.13 | 0.90 | 3.00 | 2.00 |
|  |  | Urgency | 3.67 | 0.92 | 4.00 | 1.00 |  | 3.67 | 0.82 | 4.00 | 1.00 |
|  |  | Priority | 3.67 | 1.01 | 4.00 | 1.00 |  | 4.00 | 0.66 | 4.00 | 0.00 |
| Organizational | Antimicrobial stewardship | | |  |  |  |  |  |  |  |  |
|  |  | Contribution | 3.00 | 1.14 | 3.00 | 2.00 |  |  |  |  |  |
|  |  | Dissemination | 3.00 | 0.98 | 3.00 | 1.75 |  | 3.04 | 0.46 | 3.00 | 0.00 |
|  |  | Impact | 4.25 | 0.53 | 4.00 | 1.00 |  | 3.88 | 0.68 | 4.00 | 0.00 |
|  |  | Cost | 2.92 | 0.93 | 3.00 | 0.75 |  | 3.08 | 0.88 | 3.00 | 1.50 |
|  |  | Urgency | 3.92 | 0.72 | 4.00 | 1.00 |  | 3.75 | 0.68 | 4.00 | 1.00 |
|  |  | Priority | 3.88 | 0.90 | 4.00 | 1.75 |  | 3.79 | 0.66 | 4.00 | 0.75 |
| Organizational | Blood and blood product management protocols | | | | |  |  |  |  |  |  |
|  |  | Contribution | 3.65 | 1.11 | 4.00 | 1.00 |  |  |  |  |  |
|  |  | Dissemination | 3.83 | 0.83 | 4.00 | 1.00 |  | 3.91 | 0.67 | 4.00 | 1.00 |
|  |  | Impact | 3.83 | 0.89 | 4.00 | 1.00 |  | 3.78 | 0.85 | 4.00 | 1.00 |
|  |  | Cost | 2.96 | 0.93 | 3.00 | 0.00 |  | 3.13 | 0.63 | 3.00 | 1.00 |
|  |  | Urgency | 3.26 | 0.96 | 3.00 | 1.00 |  | 3.43 | 0.79 | 4.00 | 1.00 |
|  |  | Priority | 3.26 | 1.10 | 3.00 | 1.00 |  | 3.48 | 0.79 | 3.00 | 1.00 |
| Organizational | Medical equipment sterilisation protocols | | | | |  |  |  |  |  |  |
|  |  | Contribution | 3.58 | 1.10 | 4.00 | 1.00 |  |  |  |  |  |
|  |  | Dissemination | 3.79 | 0.93 | 4.00 | 1.75 |  | 3.96 | 0.75 | 4.00 | 1.75 |
|  |  | Impact | 3.96 | 0.91 | 4.00 | 2.00 |  | 3.75 | 0.90 | 4.00 | 1.00 |
|  |  | Cost | 3.29 | 1.08 | 3.00 | 1.00 |  | 3.46 | 0.78 | 3.00 | 1.00 |
|  |  | Urgency | 3.29 | 0.91 | 3.00 | 1.00 |  | 3.08 | 0.78 | 3.00 | 0.75 |
|  |  | Priority | 3.42 | 1.02 | 3.00 | 1.00 |  | 3.21 | 0.83 | 3.00 | 1.00 |
| Clinical | Medication management / reconciliation protocols | | | | | |  |  |  |  |  |
|  |  | Contribution | 2.70 | 1.15 | 2.00 | 2.00 |  |  |  |  |  |
|  |  | Dissemination | 2.87 | 1.10 | 3.00 | 2.00 |  | 2.96 | 0.64 | 3.00 | 0.00 |
|  |  | Impact | 4.30 | 0.63 | 4.00 | 1.00 |  | 4.13 | 0.69 | 4.00 | 1.00 |
|  |  | Cost | 3.61 | 1.16 | 4.00 | 2.00 |  | 3.57 | 0.90 | 3.00 | 1.00 |
|  |  | Urgency | 3.96 | 0.77 | 4.00 | 0.00 |  | 3.96 | 0.71 | 4.00 | 0.00 |
|  |  | Priority | 4.17 | 0.72 | 4.00 | 1.00 |  | 4.22 | 0.74 | 4.00 | 1.00 |
| Clinical | Transcribing error systems and protocols | | | | |  |  |  |  |  |  |
|  |  | Contribution | 3.17 | 1.23 | 3.00 | 2.00 |  |  |  |  |  |
|  |  | Dissemination | 3.13 | 0.81 | 3.00 | 1.00 |  | 3.29 | 0.69 | 3.00 | 1.00 |
|  |  | Impact | 4.00 | 0.80 | 4.00 | 1.00 |  | 4.00 | 0.72 | 4.00 | 0.00 |
|  |  | Cost | 3.70 | 0.93 | 4.00 | 1.00 |  | 3.75 | 0.85 | 4.00 | 1.00 |
|  |  | Urgency | 3.57 | 0.84 | 4.00 | 1.00 |  | 3.88 | 0.80 | 4.00 | 0.00 |
|  |  | Priority | 3.70 | 0.88 | 4.00 | 1.00 |  | 3.96 | 0.69 | 4.00 | 0.00 |
| Clinical | Smart infusion pumps and drug administration systems | | | | | |  |  |  |  |  |
|  |  | Contribution | 3.04 | 1.20 | 3.00 | 2.00 |  |  |  |  |  |
|  |  | Dissemination | 2.92 | 0.93 | 3.00 | 0.75 |  | 2.92 | 0.72 | 3.00 | 0.75 |
|  |  | Impact | 4.00 | 0.83 | 4.00 | 0.75 |  | 3.67 | 0.82 | 4.00 | 1.00 |
|  |  | Cost | 4.21 | 1.02 | 4.00 | 1.00 |  | 4.33 | 0.87 | 4.50 | 1.00 |
|  |  | Urgency | 3.42 | 0.97 | 3.00 | 1.00 |  | 3.58 | 0.83 | 4.00 | 1.00 |
|  |  | Priority | 3.50 | 0.98 | 4.00 | 1.00 |  | 3.58 | 0.78 | 4.00 | 1.00 |
| Clinical | Aseptic technique protocols and barrier precautions | | | | | |  |  |  |  |  |
|  |  | Contribution | 3.78 | 1.20 | 4.00 | 2.00 |  |  |  |  |  |
|  |  | Dissemination | 3.83 | 0.83 | 4.00 | 1.00 |  | 3.79 | 0.66 | 4.00 | 0.75 |
|  |  | Impact | 3.96 | 0.82 | 4.00 | 2.00 |  | 4.00 | 0.66 | 4.00 | 0.00 |
|  |  | Cost | 3.61 | 1.03 | 4.00 | 1.00 |  | 3.96 | 0.75 | 4.00 | 0.00 |
|  |  | Urgency | 3.52 | 1.12 | 4.00 | 1.00 |  | 3.58 | 0.78 | 4.00 | 1.00 |
|  |  | Priority | 3.52 | 1.20 | 4.00 | 2.00 |  | 3.63 | 0.82 | 4.00 | 1.00 |
| Clinical | Urinary catheter use and insertion protocols | | | | |  |  |  |  |  |  |
|  |  | Contribution | 3.22 | 1.24 | 3.00 | 2.00 |  |  |  |  |  |
|  |  | Dissemination | 3.57 | 1.04 | 4.00 | 1.00 |  | 3.70 | 0.56 | 4.00 | 1.00 |
|  |  | Impact | 3.61 | 0.99 | 4.00 | 1.00 |  | 3.83 | 0.58 | 4.00 | 0.00 |
|  |  | Cost | 2.96 | 1.07 | 3.00 | 2.00 |  | 3.13 | 0.63 | 3.00 | 1.00 |
|  |  | Urgency | 3.04 | 0.82 | 3.00 | 0.00 |  | 3.17 | 0.58 | 3.00 | 1.00 |
|  |  | Priority | 3.04 | 0.93 | 3.00 | 1.00 |  | 3.04 | 0.56 | 3.00 | 0.00 |
| Clinical | Central venous catheter insertion protocols | | | | |  |  |  |  |  |  |
|  |  | Contribution | 3.54 | 1.06 | 4.00 | 1.00 |  |  |  |  |  |
|  |  | Dissemination | 3.58 | 0.78 | 4.00 | 1.00 |  | 3.50 | 0.72 | 3.00 | 1.00 |
|  |  | Impact | 4.21 | 0.66 | 4.00 | 1.00 |  | 4.00 | 0.59 | 4.00 | 0.00 |
|  |  | Cost | 3.75 | 0.90 | 4.00 | 1.00 |  | 3.63 | 0.65 | 4.00 | 1.00 |
|  |  | Urgency | 3.54 | 0.88 | 4.00 | 1.00 |  | 3.63 | 0.71 | 4.00 | 1.00 |
|  |  | Priority | 3.75 | 0.79 | 4.00 | 1.00 |  | 3.71 | 0.69 | 4.00 | 0.75 |
| Clinical | Ventilator-associated pneumonia minimisation protocols | | | | | |  |  |  |  |  |
|  |  | Contribution | 3.17 | 0.94 | 3.00 | 1.00 |  |  |  |  |  |
|  |  | Dissemination | 3.39 | 0.89 | 3.00 | 1.00 |  | 3.26 | 0.62 | 3.00 | 1.00 |
|  |  | Impact | 3.87 | 0.63 | 4.00 | 1.00 |  | 3.83 | 0.65 | 4.00 | 0.00 |
|  |  | Cost | 3.26 | 0.96 | 3.00 | 1.00 |  | 3.26 | 0.62 | 3.00 | 1.00 |
|  |  | Urgency | 3.35 | 0.98 | 3.00 | 1.00 |  | 3.35 | 0.65 | 3.00 | 1.00 |
|  |  | Priority | 3.43 | 0.90 | 3.00 | 1.00 |  | 3.52 | 0.67 | 4.00 | 1.00 |
| Clinical | Procedural / surgical checklists | | | |  |  |  |  |  |  |  |
|  |  | Contribution | 3.48 | 1.08 | 4.00 | 1.00 |  |  |  |  |  |
|  |  | Dissemination | 3.43 | 0.90 | 4.00 | 1.00 |  | 3.67 | 0.70 | 4.00 | 1.00 |
|  |  | Impact | 3.91 | 0.79 | 4.00 | 1.00 |  | 3.96 | 0.55 | 4.00 | 0.00 |
|  |  | Cost | 2.52 | 1.12 | 3.00 | 2.00 |  | 2.79 | 0.59 | 3.00 | 0.00 |
|  |  | Urgency | 3.43 | 1.04 | 4.00 | 1.00 |  | 3.63 | 0.58 | 4.00 | 1.00 |
|  |  | Priority | 3.74 | 1.01 | 4.00 | 1.00 |  | 3.79 | 0.59 | 4.00 | 0.00 |
| Clinical | Operating room integration and display technology | | | | | |  |  |  |  |  |
|  |  | Contribution | 2.92 | 1.18 | 3.00 | 2.00 |  |  |  |  |  |
|  |  | Dissemination | 2.75 | 0.85 | 3.00 | 1.00 |  | 2.83 | 0.56 | 3.00 | 0.75 |
|  |  | Impact | 3.96 | 0.75 | 4.00 | 0.00 |  | 3.75 | 0.61 | 4.00 | 0.75 |
|  |  | Cost | 3.50 | 1.38 | 3.50 | 2.00 |  | 3.67 | 0.96 | 3.50 | 1.75 |
|  |  | Urgency | 3.50 | 0.98 | 4.00 | 1.00 |  | 3.46 | 0.66 | 3.00 | 1.00 |
|  |  | Priority | 3.54 | 0.98 | 3.50 | 1.00 |  | 3.46 | 0.66 | 4.00 | 1.00 |
| Clinical | Peri-operative medication protocols | | | |  |  |  |  |  |  |  |
|  |  | Contribution | 3.13 | 1.06 | 3.00 | 2.00 |  |  |  |  |  |
|  |  | Dissemination | 3.26 | 0.86 | 3.00 | 1.00 |  | 3.17 | 0.65 | 3.00 | 1.00 |
|  |  | Impact | 4.04 | 0.71 | 4.00 | 0.00 |  | 3.87 | 0.55 | 4.00 | 0.00 |
|  |  | Cost | 2.65 | 1.15 | 3.00 | 2.00 |  | 2.91 | 0.73 | 3.00 | 0.00 |
|  |  | Urgency | 3.78 | 0.95 | 4.00 | 1.00 |  | 3.65 | 0.57 | 4.00 | 1.00 |
|  |  | Priority | 3.74 | 1.01 | 4.00 | 1.00 |  | 3.74 | 0.54 | 4.00 | 0.00 |
| Clinical | Venous thromboembolism (VTE) prevention protocols | | | | | |  |  |  |  |  |
|  |  | Contribution | 3.39 | 1.16 | 3.00 | 1.00 |  |  |  |  |  |
|  |  | Dissemination | 3.52 | 0.85 | 4.00 | 1.00 |  | 3.70 | 0.56 | 4.00 | 1.00 |
|  |  | Impact | 4.00 | 0.67 | 4.00 | 0.00 |  | 3.96 | 0.47 | 4.00 | 0.00 |
|  |  | Cost | 3.26 | 0.92 | 3.00 | 1.00 |  | 3.35 | 0.65 | 3.00 | 1.00 |
|  |  | Urgency | 3.57 | 0.73 | 4.00 | 1.00 |  | 3.65 | 0.65 | 4.00 | 1.00 |
|  |  | Priority | 3.52 | 0.73 | 4.00 | 1.00 |  | 3.78 | 0.52 | 4.00 | 0.00 |
| Clinical | Clinical care standards | | |  |  |  |  |  |  |  |  |
|  |  | Contribution | 3.22 | 1.00 | 3.00 | 1.00 |  |  |  |  |  |
|  |  | Dissemination | 3.26 | 0.75 | 3.00 | 1.00 |  | 3.26 | 0.62 | 3.00 | 1.00 |
|  |  | Impact | 4.09 | 0.60 | 4.00 | 0.00 |  | 3.74 | 0.62 | 4.00 | 0.00 |
|  |  | Cost | 2.70 | 1.06 | 3.00 | 1.00 |  | 3.04 | 0.71 | 3.00 | 1.00 |
|  |  | Urgency | 3.48 | 0.85 | 4.00 | 1.00 |  | 3.61 | 0.66 | 4.00 | 1.00 |
|  |  | Priority | 3.57 | 0.79 | 4.00 | 1.00 |  | 3.65 | 0.65 | 4.00 | 1.00 |
| Clinical | Pressure injury (ulcer) prevention protocols | | | | |  |  |  |  |  |  |
|  |  | Contribution | 3.35 | 0.98 | 3.00 | 1.00 |  |  |  |  |  |
|  |  | Dissemination | 3.83 | 0.83 | 4.00 | 1.00 |  | 3.70 | 0.47 | 4.00 | 1.00 |
|  |  | Impact | 3.74 | 0.75 | 4.00 | 1.00 |  | 3.65 | 0.71 | 4.00 | 1.00 |
|  |  | Cost | 2.78 | 1.00 | 3.00 | 1.00 |  | 3.13 | 0.55 | 3.00 | 0.00 |
|  |  | Urgency | 2.87 | 0.92 | 3.00 | 1.00 |  | 3.17 | 0.65 | 3.00 | 1.00 |
|  |  | Priority | 3.04 | 0.98 | 3.00 | 2.00 |  | 3.17 | 0.65 | 3.00 | 1.00 |
| Clinical | Falls prevention initiatives | | |  |  |  |  |  |  |  |  |
|  |  | Contribution | 3.14 | 1.08 | 3.00 | 1.25 |  |  |  |  |  |
|  |  | Dissemination | 3.68 | 0.89 | 4.00 | 1.00 |  | 3.57 | 0.79 | 4.00 | 1.00 |
|  |  | Impact | 3.36 | 0.95 | 3.00 | 1.00 |  | 3.48 | 0.73 | 3.00 | 1.00 |
|  |  | Cost | 3.68 | 1.17 | 4.00 | 2.00 |  | 3.57 | 0.66 | 4.00 | 1.00 |
|  |  | Urgency | 3.45 | 1.14 | 4.00 | 1.25 |  | 3.26 | 0.62 | 3.00 | 1.00 |
|  |  | Priority | 3.41 | 1.01 | 3.50 | 1.25 |  | 3.43 | 0.66 | 3.00 | 1.00 |
| Clinical | Acute delirium & cognitive impairment management initiatives | | | | | | | |  |  |  |
|  |  | Contribution | 2.77 | 0.87 | 3.00 | 1.00 |  |  |  |  |  |
|  |  | Dissemination | 2.91 | 0.87 | 3.00 | 1.25 |  | 2.70 | 0.63 | 3.00 | 1.00 |
|  |  | Impact | 3.68 | 0.84 | 4.00 | 1.00 |  | 3.65 | 0.65 | 4.00 | 1.00 |
|  |  | Cost | 3.18 | 1.01 | 3.00 | 1.25 |  | 3.00 | 0.60 | 3.00 | 0.00 |
|  |  | Urgency | 3.91 | 0.92 | 4.00 | 2.00 |  | 3.83 | 0.65 | 4.00 | 0.00 |
|  |  | Priority | 3.73 | 1.03 | 4.00 | 1.25 |  | 3.91 | 0.60 | 4.00 | 0.00 |
| Clinical | Response to clinical deterioration | | | |  |  |  |  |  |  |  |
|  |  | Contribution | 2.86 | 0.83 | 3.00 | 1.25 |  |  |  |  |  |
|  |  | Dissemination | 3.05 | 1.05 | 3.00 | 2.00 |  | 2.87 | 0.55 | 3.00 | 0.00 |
|  |  | Impact | 4.00 | 0.69 | 4.00 | 0.50 |  | 3.83 | 0.49 | 4.00 | 0.00 |
|  |  | Cost | 3.77 | 1.07 | 4.00 | 2.00 |  | 3.48 | 0.79 | 3.00 | 1.00 |
|  |  | Urgency | 3.68 | 0.95 | 4.00 | 1.00 |  | 3.91 | 0.51 | 4.00 | 0.00 |
|  |  | Priority | 3.68 | 0.95 | 4.00 | 1.00 |  | 3.91 | 0.51 | 4.00 | 0.00 |
| Clinical | Patient hydration and nutrition standards | | | | |  |  |  |  |  |  |
|  |  | Contribution | 2.77 | 0.75 | 3.00 | 1.00 |  |  |  |  |  |
|  |  | Dissemination | 3.09 | 0.81 | 3.00 | 1.25 |  | 3.13 | 0.34 | 3.00 | 0.00 |
|  |  | Impact | 3.50 | 0.67 | 3.00 | 1.00 |  | 3.13 | 0.46 | 3.00 | 0.00 |
|  |  | Cost | 2.73 | 0.63 | 3.00 | 1.00 |  | 2.83 | 0.39 | 3.00 | 0.00 |
|  |  | Urgency | 3.09 | 0.75 | 3.00 | 0.25 |  | 3.04 | 0.47 | 3.00 | 0.00 |
|  |  | Priority | 3.27 | 0.77 | 3.00 | 1.00 |  | 3.04 | 0.47 | 3.00 | 0.00 |
| Clinical | Patient identification and procedure matching protocols | | | | | |  |  |  |  |  |
|  |  | Contribution | 3.55 | 1.01 | 4.00 | 1.00 |  |  |  |  |  |
|  |  | Dissemination | 3.82 | 0.80 | 4.00 | 1.00 |  | 3.96 | 0.62 | 4.00 | 0.00 |
|  |  | Impact | 3.73 | 0.94 | 4.00 | 1.25 |  | 3.92 | 0.65 | 4.00 | 0.00 |
|  |  | Cost | 2.18 | 1.01 | 2.00 | 2.00 |  | 2.54 | 0.98 | 2.50 | 1.00 |
|  |  | Urgency | 3.41 | 0.96 | 3.50 | 1.00 |  | 3.75 | 0.79 | 4.00 | 1.00 |
|  |  | Priority | 3.45 | 1.01 | 3.50 | 1.00 |  | 3.79 | 0.72 | 4.00 | 0.00 |
